# Simultaneous Parameters Identifiability and Estimation of an *E. coli* Metabolic Network Model

**DOI:** 10.1155/2015/454765

**Published:** 2015-01-06

**Authors:** Kese Pontes Freitas Alberton, André Luís Alberton, Jimena Andrea Di Maggio, Vanina Gisela Estrada, María Soledad Díaz, Argimiro Resende Secchi

**Affiliations:** ^1^Programa de Engenharia Química-COPPE, Universidade Federal do Rio de Janeiro, Cidade Universitária, 21941-972 Rio de Janeiro, BR, Brazil; ^2^Instituto de Química, Universidade do Estado do Rio de Janeiro, São Francisco Xavier 524, 20550-900 Rio de Janeiro, BR, Brazil; ^3^Planta Piloto de Ingeniería Química-CONICET, Universidad Nacional del Sur, Camino La Carrindanga, Km 7, 8000 Bahía Blanca, Argentina

## Abstract

This work proposes a procedure for simultaneous parameters identifiability and estimation in metabolic networks in order to overcome difficulties associated with lack of experimental data and large number of parameters, a common scenario in the modeling of such systems. As case study, the complex real problem of parameters identifiability of the *Escherichia coli* K-12 W3110 dynamic model was investigated, composed by 18 differential ordinary equations and 35 kinetic rates, containing 125 parameters. With the procedure, model fit was improved for most of the measured metabolites, achieving 58 parameters estimated, including 5 unknown initial conditions. The results indicate that simultaneous parameters identifiability and estimation approach in metabolic networks is appealing, since model fit to the most of measured metabolites was possible even when important measures of intracellular metabolites and good initial estimates of parameters are not available.

## 1. Introduction

The development of mathematical model for metabolic networks has been severely hampered by the lack of kinetic information [1–4]. Usually, available experimental data are obtained under different conditions using heterogeneous techniques, whose choice must be done according to the observation of a specific phenomenon of interest on the pathways [2, 4–6]. In such systems, the type of experiment, sampling method, and the mathematical interpretation of the data depend on the desired experimental information [5]. However, as pointed out by Costa et al. [4], kinetic information presented in the literature about metabolic network models is scarce and often confuse; thus, other strategies are adopted in detriment to the dynamic simulation of such systems.

Mathematically, metabolic networks are described by complex dynamics models, whose structure is composed by ordinary differential equations that represent mass balance of the substrate, biomass, products and intracellular metabolites crucial on the pathways, and numerous reaction rates regarding to the pathways. Such mathematical structure presents a large number of parameters, for which the estimation procedure demands a considerable number of experimental data. Since experimentally metabolic networks are only partially observed, only a fraction of the intracellular metabolites considered in the mathematical model can be directly measured and thus initial conditions should also be estimated. Unfortunately, in metabolic network systems, lack of experimental data is almost unavoidable, which compromises the reliability of reactions rates proposition and makes the estimation of all parameters unfeasible. Thus, such problems often require the use of parameters identifiability procedures.

Parameters identifiability procedures deal with ill-posed parameters estimation problems, selecting a subset of parameters that can be estimated when the estimation of all parameters is not possible [7]. In most procedures, parameters are ranked from most estimable to least estimable based on the structure of the model, the experimental measurements and their uncertainties, and the uncertainties of the initial estimates [8].

Several studies reported in the literature addressing the parameters identifiability in metabolic networks are exclusively concentrated in the model structure, called* structural identifiability* (e.g., Davidescu and Jørgensen [9]; Roper et al. [10]; Nikerel et al. [11]), and do not take into account the available experimental data. On other side, the* practical identifiability* (e.g., Srinath and Gunawan [12]) investigates if the available experimental data are appropriate and sufficient for achieving a reliably estimation of the model parameters.

Although the structural identifiability is a necessary condition, the practical identifiability must overcome additional difficulties, including the selection of parameters with low sensitivity on the model predictions and correlation among parameters [13]. Unfortunately, such analyses in complex models depend on the values of parameters [13], generally unavailable [8].

Since sensitivity analysis is a key tool of identifiability procedures, procedures that evaluate the identifiability only based on initial parameters values can lead to a subset of selected parameters whose estimation may lead to an ill-posed problem [7]. A strategy to soften this problem is to perform simultaneous parameters selection and estimation, which ensures the estimation of the selected parameters (e.g., Secchi et al. [14], Wu et al. [15]; Wu et al. [16]; McLean et al. [17]; Alberton et al. [18]).

Several procedures adopt as stop criterion the singularity of FIM (Fisher Information Matrix) (e.g., Weijers and Vanrolleghem [19]; Sandink et al. [20]; Li et al. [21]; Secchi et al. [14]; Lund and Foss [22]; Thompson et al. [8]; Alberton et al. [18]). The singularity of the FIM matrix, calculated only with the selected parameters, indicates the point in which estimation problem becomes ill-posed. When such point is reached, the parameters selection is stopped and the remaining parameters are admitted as nonidentifiable parameters. Particularly when identifiability performs simultaneous parameters selection and estimation (e.g., Secchi et al. [14]; Wu et al. [15]; Wu et al. [16]; McLean et al. [17]; Alberton et al. [18]), it is not desirable to keep the remaining parameters as nonidentifiable without evaluation of their estimation potential, because the estimation problem is modified at each selected parameter.

A great challenge to be overcame in identifiability procedures, even those which include simultaneous estimation, is that the nonselected parameters are evaluated based on their initial estimates, which are probably inadequate. The literature addresses Monte Carlo techniques [23] and simultaneous parameters reestimation for assuring well posed estimation of the selected parameters (e.g., Secchi et al. [14], Wu et al. [15]; Wu et al. [16]; McLean et al. [17]; Alberton et al. [18]). As more proper, the evaluation of subsequent parameters to be selected should be done based on the reestimated selected parameters values [17, 18], reducing the dependence on the initial parameters estimates. In an interesting work, McLean et al. [17] developed an algorithm which allows evaluating the identifiability of all parameters of the model; such procedure reestimate selected parameters and use these reestimated values in the selection of subsequent parameters to be evaluated, with an intensive computational efforts. Also, in the work of Alberton et al. [18], the reestimated values are used in the selection of subsequent parameters to be evaluated, but in such procedure the numerical efforts are significantly reduced using a binary search based algorithm.

Another challenge is that, even when good initial estimates of parameters values are available, in complex models the verification for identifiability problems (e.g., nonsignificant parameters or parameters correlation derived from experimental design) is a conceptual and numerical arduous task.

In such scenario, an important question to be answered is how to reduce the dependence of identifiability procedure with the initial estimates of parameters values and the selection criteria adopted? In this context, this work presents a numerical procedure for treating estimation problems present in metabolic networks based on intensive parameters evaluation that includes simultaneous parameters selection and estimation. As the main characteristic, the numerical procedure is able to investigate the identifiability of all parameters of the mathematical model, even in ill-posed estimation problem. As in Alberton et al. [18], the numerical procedure could be adapted to procedures proposed in literature for ranking parameters according to their estimability (e.g., Weijers and Vanrolleghem [19]; Sandink et al. [20]; Brun et al. [24]; Yao et al. [25]; Li et al. [21]; Secchi et al. [14]; Chu and Hahn [23]; Sun and Hahn [26]; Lund and Foss [22]; Chu et al. [27]). A complex dynamic model of the microorganism* Escherichia coli* K-12 W3110 metabolic pathways [2] illustrates the performance of the proposed numerical procedure in applications of interest. Such microorganisms are very important in bioengineering and industrial microbiology, being widely employed in processes of recombinant proteins production.

## 2. Theoretical Backgrounds

A brief description of parameters estimation and identifiability procedures is given below.

### 2.1. Parameters Estimation

Parameters estimation is achieved by minimizing an objective function, which is a measure between the difference of the predicted model outputs and experimental measurements. Parameters values can be obtained according to maximum likelihood principle, as extensively described in literature [28, 29]. Assuming that the model is perfect, experiments are well done, experimental errors follow normal distribution, and independent variables are known with high accuracy, then the parameters can be estimated, according to the maximum likelihood principle, by minimizing the following objective function [28, 29]:

(1) $$\begin{array}{c} S\left(\theta \right)={\left(Y^{C}-Y^{E}\right)}^{T}{\left(V_{Y}\right)}^{-1}\left(Y^{C}-Y^{E}\right), \end{array}$$

in which *V*_*Y*_ represents the experimental error covariance matrix, *Y* ^*E*^ is the vector of experimental data, and *Y* ^*C*^ is the vector of model predicted values. Generally, only the terms of the diagonal of the matrix *V*_*Y*_ are considered, due to the difficulties to characterize experimental errors; thus, the objective function becomes the weighted least square function.

Once the parameters have been obtained, one can determine the uncertainties in the parameters and prediction. Usually the parameters uncertainty is based on the parameters covariance matrix (*V*_Θ_), which under some simplifying assumptions contains geometrics characteristics of the confidence region of the parameters. The terms along the diagonal of the parameters covariance matrix represent the variability of the parameters estimates, and off-diagonal terms indicate the interactions among the parameters. In the parameters estimation procedure, first the Fisher information matrix (FIM) is computed and, subsequently, *V*_Θ_ as follows [28, 29]:

(2) $$\begin{array}{c} \text{FIM}={\left(\frac{\partial Y}{\partial \Theta }\right)}^{T}{\left(V_{Y}\right)}^{-1}\frac{\partial Y}{\partial \Theta },\\ V_{\Theta }={\left(\text{FIM}\right)}^{-1}, \end{array}$$

in which ∂*Y*/∂Θ represents the local sensitivity matrix *B* [28, 29].

### 2.2. Parameters Identifiability

Estimation of all parameters values may not be possible when unsatisfactory quantity and/or quality of experimental data are available or when bad model structure and/or inadequate design of experiments were built, leading to nonsignificant or high-correlated parameters with influence on model prediction.

A common approach to overcome this problem is the use of parameters identifiability, also known as parameters estimability [7, 8]. Based on structural model and available experimental data, parameters identifiability procedures partition the original set of parameters into two subsets: (i) the parameters that can be estimated, called identifiable parameters, and (ii) the parameters that cannot be estimated, called nonidentifiable parameters. In most procedures, the identifiable parameters are ranked from most estimable to least estimable and such parameters are estimated, while the nonidentifiable parameters are kept at their initial estimates. Thus, the comparison with the model fit before and after applying the identifiability procedure is verified by the improvement achieved with the selected parameters reestimation.

**Figure 1:**
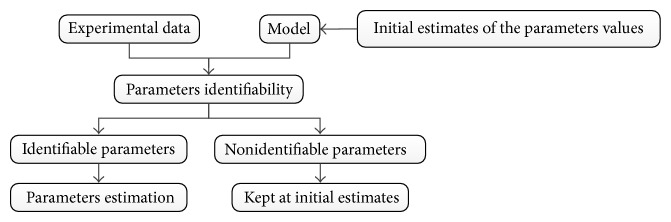
Classical scheme of parameters identifiability procedures.

A classical scheme employed by parameters identifiability procedures is showed in Figure 1. Note that in the classical scheme, the parameters estimation is carried out after the procedure; thus the quality of the initial estimates of parameters values is fundamental for a suitable selection [18].

## 3. Numerical Procedure: Intensive Parameters Evaluation

An important aspect of the classical identifiability procedures is to keep the nonidentifiable parameters in their initial estimates while the identifiable parameters are estimated. Since estimation of all parameters is not possible, the estimation of the most identifiable parameters can both regularize an ill-posed estimation problem and simplify the associated optimization problem [7]. Nonetheless, when parameters selection in the classical procedure reached the stop criteria, the remaining parameters are admitted as nonidentifiable. However, it should be important to verify if the inclusion of any other parameter is possible, in order to give the chance of all parameters to be tested. It is especially important because the parameters reestimation of the selected subset may change all parameters values. Thus, changing parameters values may allow the inclusion of other parameters, even those that has already been tested, without success. Thus, identifiability procedures with simultaneous parameters reestimation will require a high computational cost. For example, in the procedure proposed by McLean et al. [17] [(*nP*(*nP* + 1)/2) − 1] evaluations are necessary.

**Figure 2:**
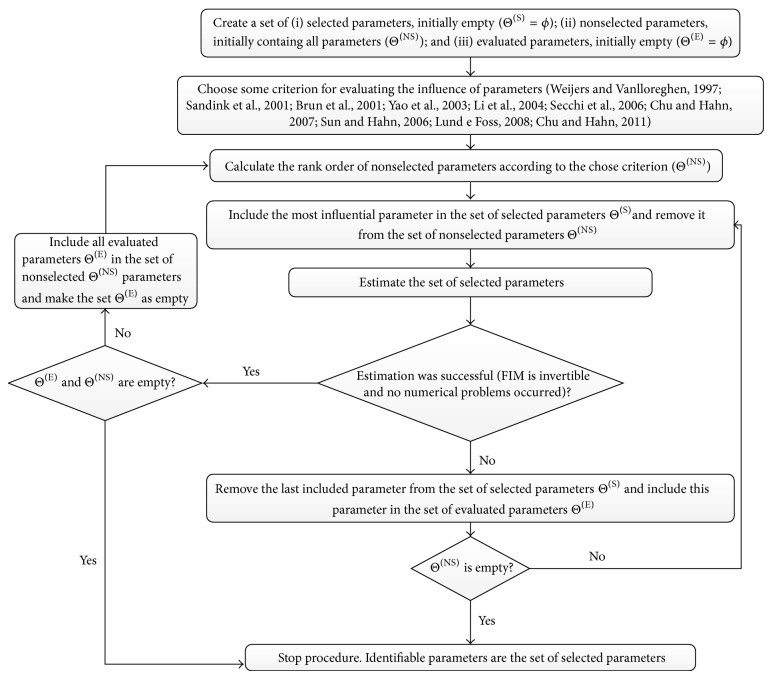
Numerical procedure proposed for parameter identifiability in metabolic networks.

The proposed numerical procedure performs intensive parameters evaluation regarding the identifiability and performs the simultaneous estimation approach, based on the one-by-one selection, as presented in Figure 2. The intensive parameters evaluation presents the following basics steps: (i) rank all parameters of the model, (ii) select the most identifiable parameter, and (iii) reestimate the set of selected parameters. If step (iii) is not successfully performed, an additional step is introduced: (iv) remove the last evaluated parameter and repeat steps (ii) and (iii) taking the next most identifiable parameter, until step (iii) is successfully performed; such evaluation is stopped only when all parameters have been evaluated.

From of description in Figure 2, essentially three sets are created: selected parameters Θ^(S)^, nonselected parameters Θ^(NS)^, and evaluated parameters Θ^(E)^. Initially, all the parameters are included in the set of nonselected parameters Θ^(NS)^. Thus, this set of parameters is ranked according to their apparent identifiability; in this paper, according to Yao et al. [25], the apparent most estimable parameter is included in the set of selected parameters and removed from the set of nonselected parameters. A reestimation step for the selected parameters is then performed. If the estimation is successfully performed, then the nonselected parameters are ranked again and the procedure is repeated. Otherwise, if the estimation failed, or some numerical problem occurs, the last included parameter must be removed from the set of selected parameters and transferred to the set of evaluated parameters Θ^(E)^. The set of evaluated parameters Θ^(E)^ contains the parameters that were not successfully performed in the set of selected parameters. The procedure seeks to include the next apparent most estimable parameter from Θ^(NS)^. If some parameter is successfully included, then after the reestimation step, with changed values of the selected parameters, the parameters Θ^(E)^ may now become estimable; therefore, in case of succeed reestimation, all parameters in the set Θ^(E)^ are transferred to the set Θ^(NS)^ to be reevaluated in next iterations. The procedure stops when Θ^(NS)^ is empty; that is, there is no more parameter to be included in the set of selected parameters. All the parameters have also been included in the set of Θ^(S)^ or Θ^(E)^. Seeking to ensure evaluation of all parameters of the model, the proposed procedure is based on the one-by-one parameters selection that has a computational effort in the worst case given as [*nP*(*nP* + 1)/2].

The proposed numerical procedure for parameters identifiability is implemented in computational code Fortran 95. In this numerical procedure, two powerful packages of literature are employed: Dassl [30], used for solving algebraic-differential equations, and Estima and Planeja [31], used for estimate the parameters of the model; this last one adapted by Alberton et al. [18] to deal complex models with scarce experimental data.

**Figure 3:**
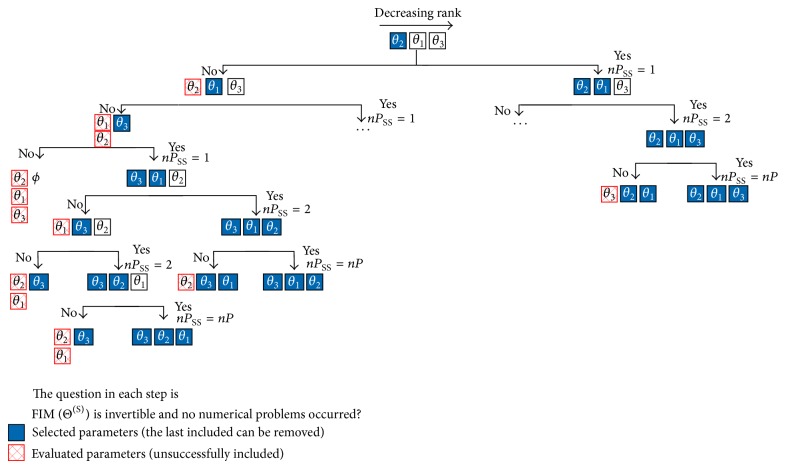
Possibilities of numerical procedure for 3 parameters investigated;* nP* and* nPSS* represent, respectively, the number of parameters and the number of succeeded selected parameters.

In order to clarify the proposed procedure, Figure 3 illustrates a case with three parameters. The parameters are ranked according to the adopted identifiability criteria (e.g., Weijers and Vanrolleghem [19]; Sandink et al. [20]; Brun et al. [24]; Yao et al. [25]; Li et al. [21]; Secchi et al. [14]; Chu and Hahn [23]; Sun and Hahn [26]; Lund and Foss [22]; Chu et al. [27]). According to the proposed procedure, the first of the most relevant parameter of the rank is included in the set of selected parameters, represented by the filled squares in Figure 3. When adding such parameter to the set of selected parameters two cases can arise: (i) the set of selected parameters can be estimated, the selected parameter was included with success; thus, the nonselected parameters are reranked and the most relevant parameter of the rank should be included in the set of selected parameters or (ii) the set of selected parameters cannot be estimated simultaneously, the last selected parameter is removed from the set of selected parameters and added to the set of evaluated parameters, represented by plaid squares in Figure 3; thus the next parameter in the identifiability rank should be selected. Since the total number of parameters is equal to 3, in the worst case the number of required evaluations is 6.

Although the proposed procedure may still be affected by the initial estimates, this dependency is strongly reduced due to the steps of parameters reestimation [18, 23]. According to Alberton et al. [18], a possible and natural alternative would be the use of nondeterministic optimization methods before or even during the identifiability procedures, such as Particle Swarm Optimization (PSO) [32] or Genetic Algorithm (GA) [33], to improve the initial parameters estimates. However, the use of nondeterministic methods may not be advantageous for complex metabolic network models, because the range of parameters values must be carefully chosen, otherwise, numerical problems associated with parameters values very often do not allow the numerical simulation of the model. Thus, for many random parameters values generated by PSO or GA, parameters estimation could not be successfully evaluated. For example, in this work, the PSO as a previous step of parameters identifiability of the* E. coli* K-12 W3110 metabolic network model was investigated, but the numerical problems associated with the model simulation did not lead to any further significant improvement regarding the initial estimates. Besides, such methods require too many function evaluations of the metabolic network, since the number of parameters to be estimated is high (131 parameters in the case study).

The Yao et al. [25] methodology was adopted as criteria for ranking the parameters according to their identifiability potential; however, it is important to emphasize that other ranking methodologies can be adopted. The Yao et al. [25] identifiability is based on the sensitivity matrix *B* and two criteria for parameters selection: (i) parameters influence on model prediction, length of sensitivity vector by Euclidian norm, and (ii) parameters correlations, linear dependence among sensitivity vectors by Gram-Schmidt orthogonalization method. Each column of matrix *B* can be understood as a sensitivity vector regarding each parameter. Generally, normalized sensitivity matrix *B*_*N*_ as shown in (3) is employed to avoid the influence of different magnitudes of variables and parameters on the sensitivity analysis.

According to Yao et al. [25], the most identifiable parameter is the one with the highest Euclidian norm of the sensitivity vector, that is, max_*j*_‖*b*_*θ*_*j*__‖. Thus, this procedure proposes to include, in the set of selected parameters, one parameter each time, according to the decreasing rank of the sensitivity vectors norms. Moreover, since the vectors can be linearly dependent, for every parameter included in the set of selected parameters, an orthogonalization procedure (Gram-Schmidt method) was performed over the matrix *B*, in order to discount the influence of linear dependence with the selected parameters

(3) $$\begin{array}{c} B_{N}=\overset{\text{Parameters}}{\overset{︷}{\underset{\text{Sensitivity}\;\text{vectors}\;\text{of}\;\text{parameters}}{\underset{︸}{\begin{array}{cccc} \theta_{1} & \theta_{2} & \cdots & \theta_{nP}\\ \left[\begin{array}{c} \frac{\partial y_{1}}{\partial \theta_{1}}\cdot \frac{\theta_{1}}{y_{1}}\\ \frac{\partial y_{2}}{\partial \theta_{1}}\cdot \frac{\theta_{1}}{y_{2}}\\ \vdots \\ \frac{\partial y_{nY}}{\partial \theta_{1}}\cdot \frac{\theta_{1}}{y_{nY}} \end{array}\right. & \begin{array}{c} \frac{\partial y_{1}}{\partial \theta_{2}}\cdot \frac{\theta_{2}}{y_{1}}\\ \frac{\partial y_{2}}{\partial \theta_{2}}\cdot \frac{\theta_{2}}{y_{2}}\\ \vdots \\ \frac{\partial y_{nY}}{\partial \theta_{2}}\cdot \frac{\theta_{2}}{y_{nY}} \end{array} & \begin{array}{c} \cdots \\ \cdots \\ \ddots \\ \cdots \end{array} & \left.\begin{array}{c} \frac{\partial y_{1}}{\partial \theta_{nP}}\cdot \frac{\theta_{nP}}{y_{1}}\\ \frac{\partial y_{2}}{\partial \theta_{nP}}\cdot \frac{\theta_{nP}}{y_{2}}\\ \vdots \\ \frac{\partial y_{nY}}{\partial \theta_{nP}}\cdot \frac{\theta_{nP}}{y_{nY}} \end{array}\right]\\ ↓ & ↓ & \cdots & ↓\\ b_{\theta_{1}} & b_{\theta_{2}} & & b_{\theta_{nP}} \end{array}}}}}\begin{array}{c} \;\left.\begin{array}{c} y_{1}\\ y_{2}\\ \underset{}{\underset{}{\vdots }}\\ \underset{}{y_{nY}} \end{array}\right\}\text{Output}\;\text{variables}. \end{array} \end{array}$$

As a previous analysis, the collinearity angle (*∠*) was evaluated among sensitivity vectors *b*_*θ*_*i*__ and *b*_*θ*_*j*__, for all parameters of the model, that is, (*b*_*θ*_*i*__, *b*_*θ*_*j*__,  *i*, *j* = 1 ⋯ *nP*), as presented in the following equation [33]:

(4) $$\begin{array}{c} {∠}_{i,j}=\text{arccos}\left(\frac{b_{\theta_{i}}^{T}b_{\theta_{j}}}{\left\|b_{\theta_{i}}\right\|\left\|b_{\theta_{j}}\right\|}\right)\;\text{with}\;{∠}_{i,j}\in \left[0,\pi \right]. \end{array}$$

As well-known from linear algebra, collinearity angles values near 90° indicate linear independence between sensitivity vectors while values near 0° or 180° indicate the opposite. It is important to emphasize that *∠* is not a rigorous analysis; correlation among parameters is only verified in parameters pars. However, this analysis can be used in a simple way to verify if correlation among pairs of parameters is expected to occur.

## 4. Application: Metabolic Networks in Large Scale Modeling

Metabolic networks models are employed for describing enzymatic activity of microorganisms. In such processes, series of sequential and parallel reactions take place, producing metabolites. Palsson [3] and Steuer and Junker [1] present detailed descriptions about the development of metabolic networks models.

Despite the great variety of computational tools available for assisting the development of such models, significant challenges are encountered in modeling living organisms and their mechanisms [34]. The development of metabolic networks models includes several steps which must interconnect with each other, related to biological knowledge, experimental data acquirement, mathematical modeling, parameters estimation, and model evaluation. A simplified scheme for construction of metabolic networks models is presented in Box 1.

Regarding step (1), Steuer and Junker [1] present several databases that can be consulted for selecting all the reactions that will be considered. Moreover, Copeland et al. [34] present computational tools employed for this step.

In step (2), there are difficulties in collecting experimental data from the literature. Besides, the experiments are time and money consuming. In fact, in many metabolic reactions, especially in the catabolic reactions and the reactions for cells energy production, turnover rates are in the range of 1.5–2.0 s^−1^. Such fast reactions make experiments with manual operation unreliable to study the dynamics of intracellular metabolite concentrations [5, 6]. This characteristic of the microsystem seriously impairs the availability of experimental data, since the choice of sampling times is crucial to the quality of information that can be obtained from experimental data.

In step (3), it is necessary to propose the set of possible reactions that may occur. The application of Topological Analysis (TA) and Flux Balance Analysis (FBA), together with experimental data [35], will lead to a stoichiometric matrix for the most relevant reactions that seems to occur. Although computational tools are available for this step [34], there are some arbitrary choices, which leads to a non-unique results.

Regarding steps (4) and (5), it is important to emphasize the possible use of databases, containing both models and initial parameters values. Databases sources are presented by Steuer and Junker [1] and Copeland et al. [34]. Nevertheless, such steps are the most difficult parts of the work, since, when not found, initial estimates of parameters may become completely arbitrary. Gerdtzen et al. [36] address the complexity at the pathway level and alert for the use of default models for biochemical processes such as the Monod/Michaelis-Menten rates with their generalization toward several substrates, reversibility, and different mechanisms of inhibition. Three major problems affect the parameters estimation of metabolic networks: (i) when experimental data do not present measures for all metabolites of interest, (ii) when measurements of some metabolites are not synchronized with other metabolic measures over the sampling time, and (iii) uncertain initial estimates of the parameters values; in many cases, good initial estimates of parameters values (or even possible ranges) are not known; thus, such values are usually arbitrarily adopted [5].

Step (6) indicates the use of parameters identifiability techniques. Since generally the experimental evidence is insufficient, the estimation of all parameters seldom will be possible [37]. Nevertheless, if parameters are set initially in reasonable values, one can identify the most influent set of parameters. It is important to emphasize that techniques allowing parameters reestimation should be preferable, as discussed in this work.

Steps (7) and (8) can be performed together. Since estimation of all parameters will generally not be possible, one interesting stop criterion is the experimental demand for obtaining additional information. Using just simulated results, one can verify if, for the optimal experimental design conditions, the expected information to be obtained will justify performing additional experiments. If it seems reasonable to perform more experiments, one should return to step (2), performing experiments at the condition indicated by the optimal experimental design techniques [38, 39].

From the uncertainties sources described above, discrepancies are expected to occur between model predictions and experimental results. Even so, the use of models can help to choose experimental regions for performing experimental tests where one can expect to achieve more information, thus, saving experimental efforts which are time and money consuming. As shown in this paper, it can be well performed for large scale metabolic networks.

### 4.1. Case Study: Mathematical Model of Metabolic Networks in Large Scale—*Escherichia coli* K-12 W3110

Possibly,* Escherichia coli* is the most studied microorganism in the literature. Even so, its metabolic networks are not completely observable. For different experimental conditions, there are several infrequent or absent measures of intracellular metabolites. Thus, some studies have focused on the development of kinetic model for metabolic network for this microorganism (e.g., Chassagnole et al. [2]; di Maggio et al. [40]; Degenring [41]; Usuda et al. [42]).

Particularly,* E. coli* K-12 W3110 to be wild type (wt) corresponds to standard strain that can be genetically manipulated allowing a large range of applications, such as: (i) pharmaceutical, recombinants proteins, vaccines, and serums, (ii) genetic medicine, (iii) environmental, biomarkers and pollution-fighting, and (v) energetic, biodiesel, oils, and others.* E. coli* is able to metabolize a large variety of components (e.g., carbohydrates, proteins, amino acids, lipids, and organics acids), produce* catalase*, and also utilize a variety of sources (e.g., glucose, ammonia, and nitrogen). Besides,* E. coli* grows in large concentrations allowing fermentative processes of high yield [43].

Regarding parameters identifiability in* E. coli*, di Maggio et al. [40] used global sensitivity analysis proposed by Sobol' [44] to determine the most influential parameters on the dynamics of the central carbon metabolism of this bacterium, based on the model proposed by Chassagnole et al. [2]. di Maggio et al. [40] concluded that twelve kinetic parameters were the most influential for the model predictions. These parameters represent maximum reaction rates, inhibition, and half-saturation constants. Their identification and later estimation provide the starting points for the manipulation of certain enzyme properties [40].

**Figure 4:**
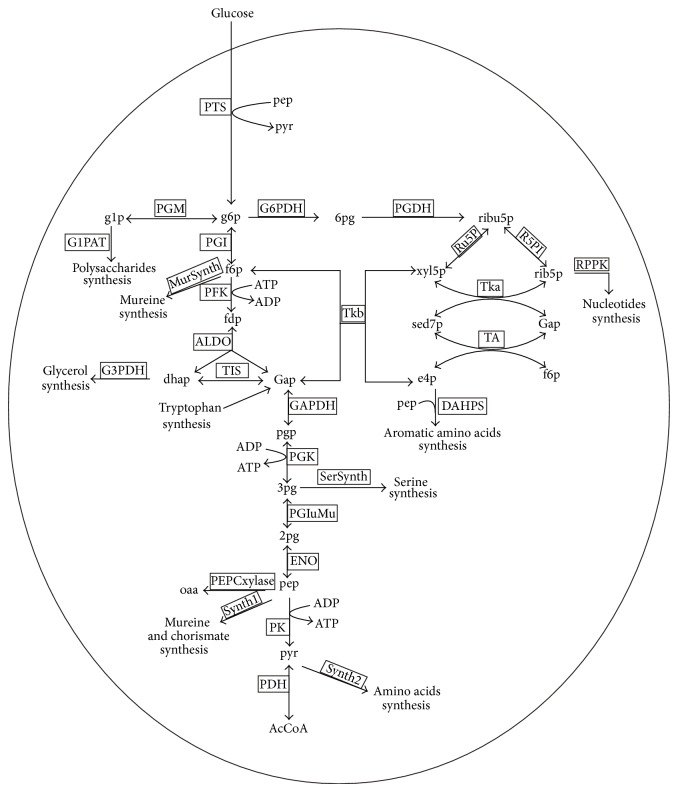
*Escherichia coli* central carbon metabolism [2].

Large scale metabolic network of the glycolysis, the pentose-phosphate-pathway, and the phosphotransferase system of* E. coli* K-12 W3110 (Figure 4) [40] consists of a complex mathematical structure with 18 dynamic mass balance equations, (5) to (22) presented below, 7 additional algebraic equations, (A.1) to (A.7), and 30 kinetic rate expressions, (A.8) to (A.37) [2]. Some enzyme kinetics modifications were introduced by di Maggio et al. [40] as the kinetic expression for the activity of phosphofructokinase was taken from Ricci [45] (see (A.12)) and the activity of glucose-6-phosphate dehydrogenase was modeled by the expression proposed by Ratushny et al. [46] (see (A.13)).

*Mass Balances to the Substrate (See (5)) and Intracellular Metabolites (See (6)–(22))*

 glucose: glc
  (5) $$\begin{array}{c} \frac{dC_{\text{glc}}^{\text{extracellular}}}{dt}=D\left(C_{\text{glc}}^{\text{feed}}-C_{\text{glc}}^{\text{extracellular}}\right)+f_{\text{pulse}}-\frac{C_{x}r_{\text{PTS}}}{\rho_{x}}, \end{array}$$
 glucose-6-phosfato: g6p
  (6) $$\begin{array}{c} \frac{dC_{\text{g}6\text{p}}}{dt}=r_{\text{PTS}}-r_{\text{PGI}}-r_{\text{G}6\text{PDH}}-r_{\text{PGM}}-\mu C_{\text{g}6\text{p}}, \end{array}$$
 fructose-6-phosphate: f6p
  (7) $$\begin{array}{c} \frac{dC_{\text{f}6\text{p}}}{dt}=r_{\text{PGI}}-r_{\text{PFK}}+r_{\text{TKb}}+r_{\text{TA}}-2r_{\text{MurSynth}}-\mu C_{\text{f}6\text{p}}, \end{array}$$
 fructose-1,6-biphosphate: fdp
  (8) $$\begin{array}{c} \frac{dC_{\text{fdp}}}{dt}=r_{\text{PKF}}-r_{\text{ALDO}}-\mu C_{\text{fdp}}, \end{array}$$
 glyceraldehyde-3-phosphate: gap
  (9) dCgap⁡dt=rALDO+rTIS−rGAPDH+rTKa+rTKb−rTA+rTrpSynth−μCgap⁡,
 dihydroxyacetonephosphate: dhap
  (10) $$\begin{array}{c} \frac{dC_{\text{dhap}}}{dt}=r_{\text{ALDO}}-r_{\text{TIS}}-r_{\text{G}3\text{PDH}}-\mu C_{\text{dhap}}, \end{array}$$
 1,3-diphosphoglycerate: pgp
  (11) $$\begin{array}{c} \frac{dC_{\text{pgp}}}{dt}=r_{\text{GAPDH}}-r_{\text{PGK}}-\mu C_{\text{pgp}}, \end{array}$$
 3-phosphoglycerate: 3pg
  (12) $$\begin{array}{c} \frac{dC_{3\text{pg}}}{dt}=r_{\text{PGK}}-r_{\text{PGGluMu}}-r_{\text{SerSynth}}-\mu C_{3\text{pg}}, \end{array}$$
 2-phosphoglycerate: 2pg
  (13) $$\begin{array}{c} \frac{dC_{2\text{pg}}}{dt}=r_{\text{PGluMu}}-r_{\text{ENO}}-\mu C_{2\text{pg}}, \end{array}$$
 phosphoenolpyruvate: pep
  (14) dCpepdt=rENO−rPK−rPTS−rPEPCxylase−rDAHPS−rSynth1−μCpep,
 pyruvate: pyr
  (15) dCpyrdt=rPK+rPTS−rPDH−rSynth2+rMetSynth+rTrpSynth−μCpyr,
 6-phosphogluconate: 6pg
  (16) $$\begin{array}{c} \frac{dC_{6\text{pg}}}{dt}=r_{\text{G}6\text{PDH}}-r_{\text{PGDH}}-\mu C_{6\text{pg}}, \end{array}$$
 ribulose-5-phosphate: ribu5p
  (17) $$\begin{array}{c} \frac{dC_{\text{ribu}5\text{p}}}{dt}=r_{\text{PGDH}}-r_{\text{Ru}5\text{P}}-r_{\text{R}5\text{PI}}-\mu C_{\text{ribu}5\text{p}}, \end{array}$$
 xylulose-5-phosphate: xyl5p
  (18) $$\begin{array}{c} \frac{dC_{\text{xyl}5\text{p}}}{dt}=r_{\text{Ru}5\text{P}}-r_{\text{TKa}}-r_{\text{TKb}}-\mu C_{\text{xyl}5\text{p}}, \end{array}$$
 sedoheptulose-7-phosphate: sed7p
  (19) $$\begin{array}{c} \frac{dC_{\text{sed}7\text{p}}}{dt}=r_{\text{TKa}}-r_{\text{TA}}-\mu C_{\text{sed}7\text{p}}, \end{array}$$
 ribose-5-phosphate: rib5p
  (20) $$\begin{array}{c} \frac{dC_{\text{rib}5\text{p}}}{dt}=r_{\text{R}5\text{PI}}-r_{\text{TKa}}-r_{\text{RPPK}}-\mu C_{\text{rib}5\text{p}}, \end{array}$$
 erythrose-4-phosphate: e4p
  (21) $$\begin{array}{c} \frac{dC_{\text{e}4\text{p}}}{dt}=r_{\text{TA}}-r_{\text{TKb}}-r_{\text{DAHPS}}-\mu C_{\text{e}4\text{p}}, \end{array}$$
 glucose-1-phosphate: g1p
  (22) $$\begin{array}{c} \frac{dC_{\text{glp}}}{dt}=r_{\text{PGM}}-r_{\text{GIPAT}}-\mu C_{\text{glp}}. \end{array}$$

As usual, the values of initial estimates used here were found in the literature [40]. Experimental data employed in this work were obtained in Hoque et al. [43] throughout a time horizon of 300 seconds. Temporal profiles for glucose (glc), dihydroxyacetonephosphate (dhap), erythrose-4-phosphate (e4p), pyruvate (pyr), fructose-1,6-diphosphate (fdp), ribose-5-phosphate (rib5p), ribulose-5-phosphate (ribu5p), 2-phosphoglycerate (2pg), phosphoenolpyruvate (pep), glyceraldehyde-3-phosphate (gap), glucose-6-phosphate (g6p), fructose-6-phosphate (f6p), and 6-phosphogluconate (6pg) were used for parameters identifiability.

**Figure 5:**
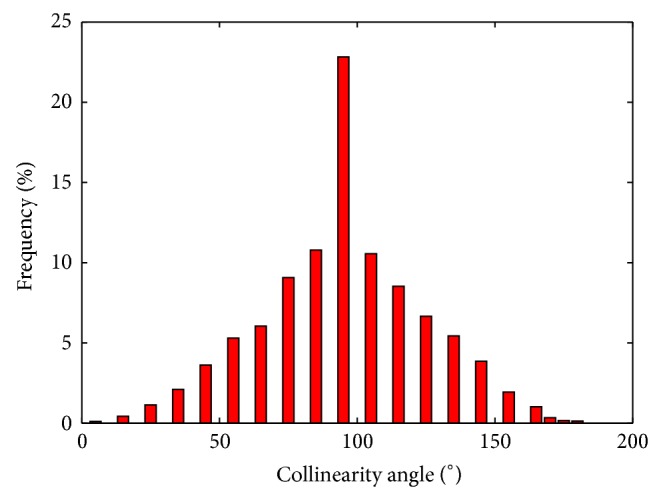
Collinearity angles among sensitivity vectors *b*_*θ*_*i*__ and *b*_*θ*_*j*__ for all parameters of the* E. coli* K-12 W3110 metabolic networks.

As a previous analysis, the collinearity angle among sensitivity vectors for all parameters of the model were calculated, and the frequency of each angle interval is shown in Figure 5. The results indicate high frequency of parameters pars linearly independents (collinearity angles values approximately 90°), in this way Table 1 presents few parameters pars with critical collinearity angles values that do not expect to be selected until a first successfully performed selection.

Fifty eight parameters have been identified, together with five initial conditions of metabolites concentrations which were not known from experimental data (pgp = 1.215 × 10^−5^, 3pg = 1.911, xyl5p = 1.564, sed7p = 4.085 × 10^−2^, and g1p = 6.500 × 10^−2^). In metabolic networks, the initial conditions of intracellular metabolites are very important. It is not recommended to keep initial conditions of unknown metabolites at zero, because numerical problems in model integration can occur or predicted values can become unreliable. In such situations, the initial concentrations of unknown metabolites must be estimated together with the parameters. In order to obtain a first initial estimates of nonmeasured intracellular metabolites, the following procedures were employed in this paper: (i) initially, keep the initial concentrations of nonmeasured intracellular metabolites at zero and simulate the model until next integration point (first numerical integration step must be small), and (ii) take the calculated values for the nonmeasured intracellular metabolites concentrations as initial conditions; the information obtained in (ii) is given as initial estimates of concentrations of nonmeasured intracellular metabolites in the identifiability procedure.

**Figure 6:**
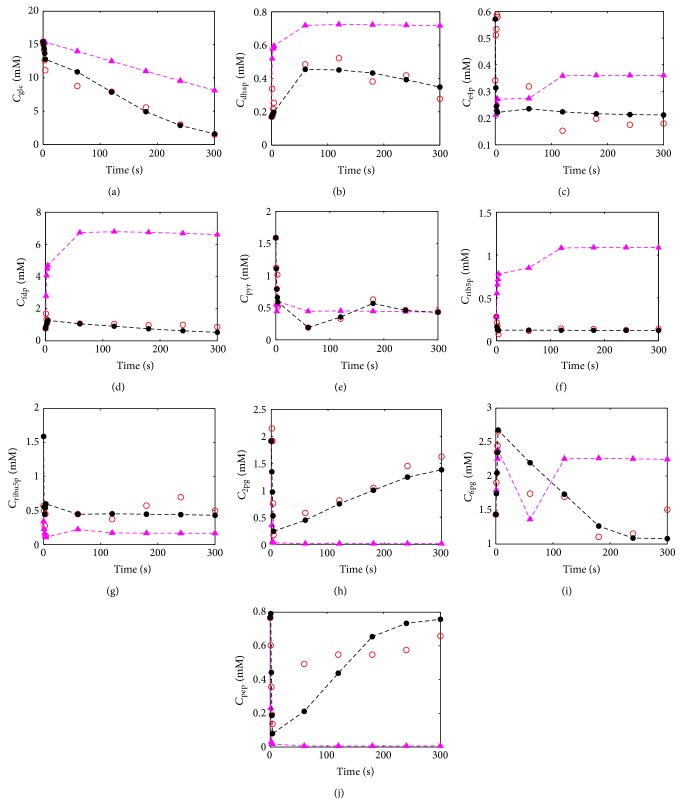
Experimental and predicted metabolites concentrations as function of the time: (○) experimental value, (-**▲**-) predicted value using initial estimates, and (-●-) predicted value after parameter identifiability using model of* E. coli* K-12 W3110 metabolic networks.

Numerical results for the proposed procedure are presented in Figure 6 and Table 2, comparing the model simulation with initial parameters estimates from the literature [40] and reestimated parameters according to the intensive parameters evaluation.

Figure 6 demonstrates the improvement of the model prediction by using the proposed procedure of parameters identifiability. Unfortunately, model limitations and few experimental data impose some insurmountable barriers to identifiability procedures. Thus, some metabolites did not change after applying the identifiability procedure. In this way, the 10 metabolites profiles that were significantly improved after the identifiability procedure are presented. Since the model fits reasonably the experimental data, it can be concluded that the parameters selection was performed with relatively good success. A special attention should be given to Figures 6(b), 6(d), and 6(f) for which the mathematical model behavior using initial estimates of parameters values is not able to follow the tendency of the experimental data. For these cases, the initial estimates of parameters values showed to be inadequate.

Table 2 presents the estimated parameters together with the initial estimates and their normalized standard deviations, calculated as the ratio between the standard deviation of the parameter and its estimated value (*σ*_*θ*_/*θ*). As important information of the identifiable parameters, the low normalized relative deviations obtained after the numerical procedure indicates a good quality of the estimated values. Analyzing the parameters estimated values, it is found that initial estimates of the parameters were significantly improved. It is important to emphasize that the estimates of the selected parameters can be significantly influenced by the initial estimates of the nonselected parameters. Thus, more adequate parameters values can be obtained when a large experimental dataset is available, allowing estimating all parameters of the mathematical model. Using the proposed numerical procedure, the objective function was also improved in two orders of magnitude relative to the initial estimates. The results are also dependent on the assumed behavior of experimental uncertainties. It is usual to assume the standard deviation of the dependent variable (*σ*_*y*_*i*__) to be proportional to its value (*y*_*i*_) in the form

(23) $$\begin{array}{c} \sigma_{y_{i}}=a\cdot y_{i}+b, \end{array}$$

where *a* and *b* are generally arbitrarily chosen. For example, for *a* = 1 × 10^−1^ and *b* = 1 × 10^−5^, the initial value of the objective function was 1.56 × 10^4^ and reduced to 3.06 × 10^3^ after reestimation. For *a* = 1 × 10^−2^ and *b* = 1 × 10^−6^, the initial value of the objective function was 3.89 × 10^7^ and reduced to 3.06 × 10^5^ after parameters reestimation. The set of selected parameters was not the same in both cases; nevertheless, the prediction was similar. The dependence of parameters estimation performance on the assumed experimental uncertainties is expected even for well posed problems. It is important information, but generally it is neglected and arbitrarily chosen due to the difficulties involved in characterizing the experimental errors.

Even with so much uncertainties, associated to experimental data, modeling development and some unknown initial conditions, it has been shown that it is possible to fit and/or improve model predictions with parameters identifiability procedures. The model can now be used for predicting the experimental behavior of the system. Besides its uncertainties, it can help to give us an idea about what we should expect in experimental regions, delimiting experimental design for further investigations, among other purposes. Naturally, for more accurate results, some predictions should be confirmed by additional experiments in the experimental regions of greater interest.

Comparing the performance of the proposed numerical procedure with similar procedure but stopping the selection when the last selected parameter was not successfully included, only 63 parameters have been selected, and the objective function was around 5 times greater compared with the results obtained with the algorithm presented in Figure 2. It emphasizes the necessity to investigate all parameters in order to improve final results and not to stop the procedure when the first parameter have failed to be included.

In the procedure proposed in previous work [18], subsets of parameters were included at once, and if the estimation of such subset was possible, this subset could not leave the set of selected parameters. Thus, if a set of parameters has been successfully estimated, the orthogonalization step (for discount parameters influence one each other) was not performed between such parameters. In the procedure presented in this work, one parameter is selected each time, and orthogonalization step between the selected parameters and yet unselected parameters is performed much more times than in previous work [18]. Although not assured by the model nonlinearities, one can expect that the present procedure should lead to better results, despite being more time consuming, since it performs a more thorough investigation regarding the parameters correlation. In fact, the objective function obtained in this work divided by the objective function of previous work [18] was 0.15, indicating a good improvement in prediction performance.

It is important to emphasize that, for this application, the improvement in the prediction quality was observed with estimation of only half of the model parameters, being the numerical procedure performed using unknown values for some initial conditions of the dependent variables. Thus, in similar scenarios, a great merit of the parameters identifiability procedure is to select only the most influential parameters on the mathematical model that can be estimated with the available experimental data.

## 5. Conclusion

In this work, a large scale ill-posed problem of parameters estimation in metabolic networks was investigated, where experimental data are scarce and concentrations of intracellular metabolites are not completely known. A proposed procedure of intensive parameters evaluation that presents simultaneous parameters selection and estimation was successfully applied to solve this problem. Compared in pairs, few parameters seem to be correlated, as revealed by collinearity angles. From the initial scenario, containing 131 parameters and 5 unknown initial conditions of intracellular metabolites concentrations, the procedure was able to identify 58 parameters, together with the 5 initial conditions of intracellular metabolites concentrations. The simultaneous reestimation step reduced the dependence on the initial parameters estimates, allowing a good fit of the model. The robustness of the applied procedure is certainly an appealing feature for metabolic networks problems.

## Figures and Tables

**Box 1:**
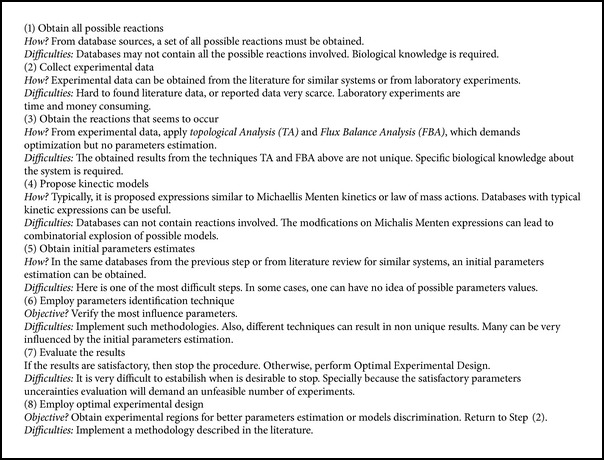
Sequence proposed for modeling metabolic systems, based on Wiechert and Graaf [35] and Steuer and Junker [1].

**Table 1:** Parameters pars of the *E. coli* K-12 W3110 metabolic networks with critical collinearity angles values.

Parameters par	Collinearity angle (°)
*K*_PTS,1_ − *K*_PTS,3_	0.5
*K*_ALDO,eq_ − *r*_TIS_^max⁡^	175.3
*K*_GAPDH,nad_ − *K*_PGK,eq_	176.5
*K*_GAPDH,eq_ − *L*_PK_	179.5
*K*_PK,amp_ − *r*_PGM_^max⁡^	176.5
*K*_GIPAT,glp_ − *K*_GIPAT,atp_	179.3
*K*_PK,amp_ − *r*_Ser,Synth_^max⁡^	2.5
*K*_PK,fdp_ − *K*_Synth1,pep_	0.0
*n*_DAHPS,pep_ − *K*_ALDO,fdp_	179.3
*K*_GAPDH,eq_ − *K*_G6PDH,tgn_	2.5
*L*_PK_ − *K*_G6PDH,tgn_	177.4
*K*_PGDH,atp_ − *K*_G1PAT,g1p_	2.4
*K*_PGDH,atp_ − *K*_G1PAT,atp_	1.6
*K*_PGDH,atp_ − *K*_PGDH,nadp_	177.5
*K*_TA,eq_ − *K*_G1PAT,g1p_	2.3
*K*_TA,eq_ − *K*_G1PAT,atp_	1.5
*K*_TA,eq_ − *K*_PGDH,atp_	0.7
*r*_PTS_^max⁡^ − *K*_G6PDH,dt_	175.2
*r*_PGDH_^max⁡^ − *K*_GAPDH,nad_	177.6

**Table 2:** Identifiable parameters of the *E. coli* K-12 W3110 metabolic networks obtained using the numerical procedure for intensive parameters evaluation.

Parameter	Initial estimate	Estimated value	Normalized standard deviation
*K*_PTS1_	3082.300	3683.300	0.060
*r*_PDH_^max⁡^	4.010	0.529	0.015
*r*_ALDO_^max⁡^	30.423	1.593	0.081
*r*_G6PDH_^max⁡^	12.873	8.476	0.041
*K*_R,pep_	750.000	0.541	0.051
*r*_GAPDH_^max⁡^	500.000	0.381	0.021
*K*_PEPCxylase,fdp_	0.700	0.469	0.028
*K*_R5P,eq_	1.400	0.647	0.064
*n*_PEPCxylase,fdp_	4.000	8.795	0.041
*r*_PK_^max⁡^	0.567	0.140	0.069
*n*_PDH_	1.000	7.237	0.028
*K*_PGI,f6p2_	0.200	1.271	0.037
*k*_tgn_	1.000	4.152	0.075
*K*_G3PDH,dhap_	1.000	19.076	0.110
*r*_TA_^max⁡^	8.461	4.051	0.051
*k*_hdt_	0.100	0.099	0.038
*K*_PGI,g6p_	0.400	0.060	0.101
*K*_PGI,f6p_	0.266	1.956 × 10^−4^	0.115
*K*_TKA,eq_	0.120	6.289	0.050
*K*_PGLUMU,2pg_	0.369	1.105	0.039
*K*_R5PI,eq_	4.000	4.182	0.052
*r*_TKA_^max⁡^	7.372	1.461	0.069
*r*_RU5PI_^max⁡^	5.182	0.100	0.054
*r*_R5PI_^max⁡^	3.733	1.366	0.019
*r*_PFK_^max⁡^	1.967	0.382	0.057
*K*_PGI,g6p2_	0.200	0.272	0.193
*L*_PK_	1.000	785.758	0.070
*K*_Synthesis1,pep_	1.000	0.361	0.053
*K*_PEPCxylase,pep_	4.070	5.731	0.038
*n*_DAHPS,e4p_	2.600	3.501	0.022
*n*_DAHPS,pep_	2.200	3.455	0.032
*e*′	0.999	1.057	0.054
*K*_T,pep_	0.750	0.630	0.122
*K*_GAPDH,nadh_	1.090	4.752	0.027
*K*_PGK,3pg_	0.473	2.326	0.077
*n*_PK_	4.000	1.582	0.030
*h*_hdt_	4.000	0.584	0.136
*K*_TIS,gap_	0.300	0.567	0.050
*h*_tgn_	2.000	9.169	0.057
*n*_G1PAT,fdp_	2.000	5.993	0.084
*E*	0.990	0.114	0.078
*K*_TA,eq_	1.050	2.583	0.054
*K*_PGLUMU,3pg_	0.200	0.084	0.035
*θ*	1.000	1.506	0.065
*K*_T,adp_	1.300	1.145	0.123
*K*_TIS,dhap_	2.800	7.830	0.057
*r*_PTS_^max⁡^	82107.310	1.876	0.070
*K*_PGLUMU,eq_	0.187	0.626	0.066
*n*_PTS,g6p_	4.000	0.443	0.037
*K*_PTS,g6p_	0.393	0.366	0.080
*K*_PKA,adp_	0.260	0.208	0.063
*K*_PKA,adp_	0.400	0.455	0.056
*K*_GAPDH,nad_	0.252	0.093	0.070
*K*_ALDO,gap2_	1.200	0.240	0.125
*K*_PK,atp_	22.500	1.483	0.062
*K*_PGDH,6pg_	5.449	3.597	0.051
*r*_PGLUMU_^max⁡^	96.972	0.749	0.063
*r*_PGDH_^max⁡^	5.221	0.597	0.036

**Table 3:** Kinetics parameters.

Enzyme	Parameter	Description
Phosphotransferase system: PTS	*K*_PTS1_	M–M half-saturation constant (mM)
	*K*_PTS2_	Constant (mM)
	*K*_PTS3_	Constant
	*K*_PTS,g6p_	Inhibition constant (mM)
	*n*_PTS,g6p_	Constant
	*r*_PTS_^max⁡^	Maximum reaction rate (mM s^−1^)

Phosphoglucoisomerase: PGI	*K*_PGI,g6p_	M–M half-saturation constant (mM)
	*K*_PGI,f6p_	Inhibition constant (mM)
	*K*_PGI,eq_	Equilibrium constant
	*K*_PGI,g6p,6pg,inh_	Inhibition constant (mM)
	*K*_PGI,f6p,6pg,inh_	Inhibition constant (mM)
	*r*_PGI_^max⁡^	Maximum reaction rate (mM s^−1^)

Phosphofructokinase: PFK	*K*_PFK,f6p,s_	M–M half-saturation constant (mM)
	*K*_PFK,atp,s_	M–M half-saturation constant (mM)
	*K*_PFK,adp,a_	Activation constant (mM)
	*K*_PFK,adp,b_	Activation constant (mM)
	*K*_PFK,adp,c_	Activation constant (mM)
	*K*_PFK,amp,a_	Activation constant (mM)
	*K*_PFK,amp,b_	Activation constant (mM)
	*K*_PFK,pep_	Inhibition constant (mM)
	*L*_PFK_	Allosteric constant
	*n*_PFK_	Number of binding sites
	*r*_PFK_^max⁡^	Maximum reaction rate (mM s^−1^)

Aldolase: ALDO	*K*_ALDO,fdp_	M–M half-saturation constant (mM)
	*K*_ALDO,dhap_	M–M half-saturation constant (mM)
	*K*_ALDO,gap_	M–M half-saturation constant (mM)
	*K*_ALDO,gap,inh_	Inhibition constant (mM)
	*V*_ALDO,blf_	Back-forward reaction rate relation
	*K*_ALDO,eq_	Equilibrium constant (mM)
	*r*_ALDO_^max⁡^	Maximum reaction rate (mM s^−1^)

Triosephosphate isomerase: TIS	*K*_TIS,dhap_	M–M half-saturation constant (mM)
	*K*_TIS,gap_	M–M half-saturation constant (mM)
	*K*_TIS,eq_	Equilibrium constant
	*r*_TIS_^max⁡^	Maximum reaction rate (mM s^−1^)

Glyceraldehyde-3-phosphate dehydrogenase: GAPDH	*K*_GAPDH,gap_	M–M half-saturation constant (mM)
	*K*_GAPDH,pgp_	Inhibition constant (mM)
	*K*_GAPDH,nad_	M–M half-saturation constant (mM)
	*K*_GAPDH,nadh_	Inhibition constant (mM)
	*K*_GAPDH,eq_	Equilibrium constant
	*r*_GAPDH_^max⁡^	Maximum reaction rate (mM s^−1^)

Phosphoglycerate kinase: PGK	*K*_PGK,pgp_	M–M half-saturation constant (mM)
	*K*_PGK,3pg_	Inhibition constant (mM)
	*K*_PGK,adp_	M–M half-saturation constant (mM)
	*K*_PGK,atp_	Inhibition constant (mM)
	*K*_PGK,eq_	Equilibrium constant
	*r*_PGK_^max⁡^	Maximum reaction rate (mM s^−1^)

Phosphoglycerate mutase: PGluMu	*K*_PGluMu,3pg_	M–M half-saturation constant (mM)
	*K*_PGluMu,2pg_	Inhibition constant (mM)
	*K*_PGluMu,eq_	Equilibrium constant
	*r*_PGluMu_^max⁡^	Maximum reaction rate (mM s^−1^)

Enolase: ENO	*K*_ENO,2pg_	M–M half-saturation constant (mM)
	*K*_ENO,pep_	Inhibition constant (mM)
	*K*_ENO,eq_	Equilibrium constant
	*r*_ENO_^max⁡^	Maximum reaction rate (mM s^−1^)

Pyruvate kinase: PK	*K*_PK,pep_	M–M half-saturation constant (mM)
	*K*_PK,adp_	M–M half-saturation constant (mM)
	*K*_PK,atp_	Inhibition constant (mM)
	*K*_PK,fdp_	Activation constant (mM)
	*K*_PK,amp_	Activation constant (mM)
	*L*_PK_	Allosteric constant
	*n*_PK_	Number of binding sites
	*r*_PK_^max⁡^	Maximum reaction rate (mM s^−1^)

Pyruvate dehydrogenase: PDH	*K*_PDH,pyr_	M–M half-saturation constant (mM)
	*n*_PDH_	Number of binding sites
	*r*_PDH_^max⁡^	Maximum reaction rate (mM s^−1^)

Phoenolpyruvate carboxylase: PEPCxylase	*K*_PEPCxylase,pep_	M–M half-saturation constant (Mm)
	*K*_PEPCxylase,fdp_	Activation constant (mM)
	*n*_PEPCxylase,fdp_	Number of binding sites
	*r*_PEPCxylase_^max⁡^	Maximum reaction rate (mM s^−1^)

Phosphoglucomutase: PGM	*K*_PGM,g6p_	M–M half-saturation constant (mM)
	*K*_PGM,g1p_	Inhibition constant (mM)
	*K*_PGM,eq_	Equilibrium constant
	*r*_PGM_^max⁡^	Maximum reaction rate (mM s^−1^)

Glucose-1-phosphate adenyltransferase: G1PAT	*K*_G1PAT,g1p_	M–M half-saturation constant (mM)
	*K*_G1PAT,atp_	M–M half-saturation constant (mM)
	*K*_G1PAT,fdp_	Activation constant (mM)
	*n*_G1PAT,fdp_	Number of binding sites
	*r*_G1PAT_^max⁡^	Maximum reaction rate (mM s^−1^)

Ribose phosphate pyrophosphokinase: RPPK	*K*_RPPK,rib5p_	M–M half-saturation constant (mM)
	*r*_RPPK_^max⁡^	Maximum reaction rate (mM s^−1^)

Glycerol-3-phosphate dehydrogenase: G3PDH	*K*_G3PDH,dhap_	M–M half-saturation constant (mM)
	*r*_G3PDH_^max⁡^	Maximum reaction rate (mM s^−1^)

Serine synthesis: SerSynth	*K*_SerSynth,3pg_	M–M half-saturation constant (mM)
	*r*_SerSynth_^max⁡^	Maximum reaction rate (mM s^−1^)

DAHP synthase: DAHPS	*K*_DAHPS,e4p_	M–M half-saturation constant (mM)
	*K*_DAHPS,pep_	M–M half-saturation constant (mM)
	*n*_DAHPS,e4p_	Number of binding sites
	*n*_DAHPS,pep_	Number of binding sites
	*r*_DAHPS_^max⁡^	Maximum reaction rate (mM s^−1^)

Glucose-6-phosphate dehydrogenase: G6PDH	*K*_G6PDH,g6p_	M–M half-saturation constant (mM)
	*K*_G6PDH,nadp_	M–M half-saturation constant (mM)
	*K*_G6PDH,nadph,nadph,inh_	Inhibition constant (mM)
	*K*_G6PDH,nadph,g6ph,inh_	Inhibition constant (mM)
	*r*_G6PDH_^max⁡^	Maximum reaction rate (mM s^−1^)

6-Phosphogluconate dehydrogenase: PGDH	*K*_PGDH,6pg_	M–M half-saturation constant (mM)
	*K*_PGDH,nadp_	M–M half-saturation constant (mM)
	*K*_PGDH,nadph,inh_	Inhibition constant (mM)
	*K*_PGDH,atp,inh_	Inhibition constant (mM)
	*r*_PGDH_^max⁡^	Maximum reaction rate (mM s^−1^)

Ribulose phosphate epimerase: RU5P	*K*_RU5P,EQ_	Equilibrium constant (mM)
	*r*_RU5PI_^max⁡^	Maximum reaction rate (mM s^−1^)

Ribose phosphate isomerase: R5PI	*K*_R5PI,eq_	Equilibrium constant (mM)
	*r*_R5PI_^max⁡^	Maximum reaction rate (mM s^−1^)

Transketolase a: TKa	*K*_TKa,eq_	Equilibrium constant (mM)
	*r*_TKa_^max⁡^	Maximum reaction rate (mM s^−1^)

Transketolase b: TKb	*K*_TKb,eq_	Equilibrium constant (mM)
	*r*_TKb_^max⁡^	Maximum reaction rate (mM s^−1^)

Transaldolase: TA	*K*_TA,eq_	Equilibrium constant (mM)
	*r*_TA_^max⁡^	Maximum reaction rate (mM s^−1^)

Synthesis 1: Synth1	*K*_Synth1,pep_	M–M half-saturation constant (mM)
	*r*_Synth1_^max⁡^	Maximum reaction rate (mM s^−1^)

Synthesis 2: Synth2	*K*_Synth2,pyr_	M–M half-saturation constant (mM)
	*r*_Synth2_^max⁡^	Maximum reaction rate (mM s^−1^)

Mureine synthesis: MurSynth	*r*_MurSynth_^max⁡^	Maximum reaction rate (mM s^−1^)

Tryptophan synthesis: TrpSynth	*r*_TrpSynth_^max⁡^	Maximum reaction rate (mM s^−1^)

Methionine synthesis: MetSynth	*r*_MetSynth_^max⁡^	Maximum reaction rate (mM s^−1^)

## Appendix

*Time Correlations for Cometabolites Concentrations [2]*

Adenosintriphosphate: atp
  (A.1) $$\begin{array}{c} C_{\text{atp}}=4.27-4.163\frac{t}{0.657+1.43t+0.364t^{2}}, \end{array}$$
adenosindiphosphate: adp
  (A.2) $$\begin{array}{c} C_{\text{adp}}=0.582+1.73\left(2.731^{-0.15t}\right)\left(0.12t+0.000214t^{3}\right), \end{array}$$
adenosin monophosphate: amp
  (A.3) Camp=0.123+7.25t7.25+1.47t+0.17t2+1.073t1.29+8.05t,
diphosphopyridine nucleotide phosphate, reduced: nadph
  (A.4) Cnadph=0.062+0.3322.718−0.46t×0.0166t1.58+0.000166t4.73  +1.16×10−10t7.89+1.36×10−13t11  +1.23×10−16t14.2,
diphosphopyridine nucleotide, reduced: nadp
  (A.5) Cnadp=0.159+0.00554t2.8+0.271t+0.01t2+0.182t4.81+0.526t,
diphosphopyridine nucleotide, reduced: nadh
  (A.6) $$\begin{array}{c} C_{\text{nadh}}=0.0934+0.0011\left(2.371^{-0.123t}\right)\left(0.844t+0.104t^{3}\right), \end{array}$$
diphosphopyridine nucleotide, oxized: nad
  (A.7) Cnad=1.314+1.3142.73−0.0435t−0.342−t+7.8712.73−0.0218t−0.1718.481+t.

*Kinetics Rates for Enzymes [2, 40].* Consider

(A.8) rPTS=rPTSmax⁡CglcextracelularCpepCpyr×1+Cg6pnPTS,g6pKPTS,g6pKPTS1+KPTS2CpepCpyr   +KPTS,3Cglcextracelular+CglcextracelularCpepCpyr   ×1+Cg6pnPTS,g6pKPTS,g6p−1,

(A.9) rPGI=rPGImax⁡Cg6p−Cf6pKPGI,eq×KPGI,g6p1+Cf6pKPGI,f6p1+C6pg/KPGI,f6p,6pginh      +C6pgKPGI,g6p,6pginh+Cg6p1+Cf6pKPGI,f6p1+C6pg/KPGI,f6p,6pginh−1,

(A.10) rPFK=rPFKmax⁡1+CpepKTpep1+CadpKTadp1+CpepKRpep1+CadpKRadpnPFKeCf6pKRf6p1+eCf6pKRf6pnPFK−11+CadpKRadpnPFK    +L01+Cpep/KTpep1+Cadp/KTadp1+Cpep/KRpep1+Cadp/KRadpnPFK    ×θce′Cf6pKRf6p1+ce′Cf6pKRf6pnPFK−11+CpepKTpep1+CadpKTadp1+CpepKRpep1+CadpKRadpnPFK ×1+Cpep/KTpep1+Cadp/KTadp1+Cpep/KRpep1+Cadp/KRadpnPFK1+eCf6pKRf6pnPFK1+CadpKRadpnPFK   +L01+Cpep/KTpep1+Cadp/KTadp1+Cpep/KRpep1+Cadp/KRadpnPFK   ×1+ce′Cf6pKRf6pCpep/KTpep1+Cpep/KTpep1+Cadp/KTadp1+Cpep/KRpep1+Cadp/KRadpnPFK−1,

(A.11) rALDO=rALDOmax⁡Cfdp−Cgap⁡CdhapKALDO,eq ×KALDO,fdp+Cfdp+KALDO,gap⁡CdhapKALDO,eqVALDO,blf   +KALDO,dhapCgap⁡KALDO,eqVALDO,blf+CfdpCgap⁡KALDO,gap⁡,inh   +CdhapCgap⁡KALDO,eqVALDO,blf−1,

(A.12) rTIS=rTISmax⁡Cdhap−Cgap⁡KTIS,eq×KTIS,dhap1+Cgap⁡KTIS,gap⁡+Cdhap−1,

(A.13) rGAPDH =rGAPDHmax⁡Cgap⁡Cnad−CpgpCnadhKGAPDH,eq  ×KGAPDH,gap⁡1+CpgpKGAPDH,pgp+Cgap⁡    ×KGAPDH,nad1+CnadhKGAPDH,nadh      +CnadKGAPDH,nad1+CnadhKGAPDH,nadh−1,

(A.14) rPGK=rPGKmax⁡CadpCpgp−CatpC3pgKPGK,eq×KPGK,adp1+CatpKPGK,atp+Cadp  ×KPGK,pgp1+C3pgKPGK,3pg+Cpgp−1,

(A.15) rPGluMu=rPGluMumax⁡C3pg−C2pgKPGluMu,eq×KPGluMu,3pg1+C2pgKPGluMu,2pg+C3pg−1,

(A.16) rENO=rENOmax⁡C2pg−CpepKENO,eq×KENO,2pg1+CpepKENO,pep+C2pg−1,

(A.17) rPK=rPKmax⁡CpepCadpCpepKPK,pep+1nPK−1 ×LPK1+Catp/KPK,atpCfdp/KPK,fdp+Camp/KPK,amp+1nPKKPK,pep    ×LPK1+Catp/KPK,atpCfdp/KPK,fdp+Camp/KPK,amp+1nPKLPKCfdpKPK,fdp+CampKPK,amp+1−11+CatpKPK,atp         ×CfdpKPK,fdp+CampKPK,amp+1−1nPK     +CpepKPK,pep+1nPK    ×Cadp+KPK,adpLPK1+Catp/KPK,atpCfdp/KPK,fdp+Camp/KPK,amp+1nPK−1,

(A.18) $$\begin{array}{c} r_{\text{PDH}}=\frac{r_{\text{PDH}}^{\max}C_{\text{pyr}}^{n_{\text{PDH}}}}{K_{\text{PDH},\text{pyr}}+C_{\text{pyr}}^{n_{\text{PDH}}}}, \end{array}$$

(A.19) rG6PDH=rGPDHmax⁡Cg6p ×1+CnadhKG6PDH,nadhhnadh1+CnadpKG6PDH,nadp1+kdtnCnadhhdt/kdthdt+Cnadhhdt+CnadphKG6PDH,nadphinh2hnadph−1KG6PDH,g6p1+ktgnCnadphtgktghtg+Cnadphtg+Cg6p   ×1+CnadphKG6PDH,nadphinh+CnadhKG6PDH,nadhinh   ×1+CnadhKG6PDH,nadhhnadh1+CnadpKG6PDH,nadp1+kdtnCnadhhdt/kdthdt+Cnadhhdt+CnadphKG6PDH,nadphinh2hnadph−1CnadpKG6PDH,nadp1+kdtn(Cnadhhdt/(kdthdt+Cnadhhdt))     ×CnadphKG6PDH,nadphinh2hnadph1+CnadpKG6PDH,nadp1+kdtnCnadhhdt/kdthdt+Cnadhhdt       +CnadphKG6PDH,nadphinh2hnadphCnadpKG6PDH,nadp1+kdtnCnadhhdt/kdthdt+Cnadhhdt     ×1+CnadhKG6PDH,nadhhnadh1+CnadpKG6PDH,nadp1+kdtnCnadhhdt/kdthdt+Cnadhhdt+CnadphKG6PDH,nadphinh2hnadph−1−1,

(A.20) rPGDH =rPGDHmax⁡C6pgCnadp  ×1+CnadphKPGDH,nadph,inh1+CatpKPGDH,atp,inhC6pg+KPGDH,6pg    ×1+CnadphKPGDH,nadph,inhCnadp+KPGDH,nadp      ×1+CnadphKPGDH,nadph,inh      ×1+CatpKPGDH,atp,inh−1,

(A.21) $$\begin{array}{c} r_{\text{R}5\text{PI}}=r_{\text{R}5\text{PI}}^{\max}\left(C_{\text{ribu}5\text{p}}-\frac{C_{\text{rib}5\text{p}}}{K_{\text{R}5\text{PI},\text{eq}}}\right), \end{array}$$

(A.22) $$\begin{array}{c} r_{\text{RU}5\text{P}}=r_{\text{RU}5\text{P}}^{\max}\left(C_{\text{ribu}5\text{p}}-\frac{C_{\text{xyl}5\text{p}}}{K_{\text{RU}5\text{P},\text{eq}}}\right), \end{array}$$

(A.23) $$\begin{array}{c} r_{\text{TKa}}=r_{\text{TKa}}^{\max}\left(C_{\text{rib}5\text{p}}C_{\text{xyl}5\text{p}}-\frac{C_{\text{sed}7\text{p}}C_{\mathrm{gap}}}{K_{\text{TKa},\text{eq}}}\right), \end{array}$$

(A.24) $$\begin{array}{c} r_{\text{TKb}}=r_{\text{TKb}}^{\max}\left(C_{\text{xyl}5\text{p}}C_{\text{e}4\text{p}}-\frac{C_{\text{f}6\text{p}}C_{\mathrm{gap}}}{K_{\text{TKb},\text{eq}}}\right), \end{array}$$

(A.25) $$\begin{array}{c} r_{\text{TA}}=r_{\text{TA}}^{\max}\left(C_{\mathrm{gap}}C_{\text{sed}7\text{p}}-\frac{C_{\text{e}4\text{p}}C_{\text{f}6\text{p}}}{K_{\text{TA},\text{eq}}}\right), \end{array}$$

(A.26) rPEPCxylase =rPEPCxylasemax⁡Cpep1+Cfdp/KPEPCxylase,fdpnPEPCxylase,fdpKPEPCxylase,pep+Cpep,

(A.27) $$\begin{array}{c} r_{\text{Synth}1}=\frac{r_{\text{Synth}1}^{\max}C_{\text{pep}}}{K_{\text{Synth}1,\text{pep}}+C_{\text{pep}}}, \end{array}$$

(A.28) $$\begin{array}{c} r_{\text{Synth}2}=\frac{r_{\text{Synth}2}^{\max}C_{\text{pyr}}}{K_{\text{Synth}2,\text{pyr}}+C_{\text{pyr}}}, \end{array}$$

(A.29) $$\begin{array}{c} r_{\text{SerSynth}}=\frac{r_{\text{SerSynth}}^{\max}C_{3\text{pg}}}{K_{\text{SerSynth},3\text{pg}}+C_{3\text{pg}}}, \end{array}$$

(A.30) $$\begin{array}{c} r_{\text{RPPK}}=\frac{r_{\text{RPPK}}^{\max}C_{\text{rib}5\text{p}}}{K_{\text{RPPK},\text{rib}5\text{p}}+C_{\text{rib}5\text{p}}}, \end{array}$$

(A.31) $$\begin{array}{c} r_{\text{G}3\text{PDH}}=\frac{r_{\text{G}3\text{PDH}}^{\max}C_{\text{dhap}}}{K_{\text{G}3\text{PDH},\text{dhap}}+C_{\text{dhap}}}, \end{array}$$

(A.32) rDAHPS =rDAHPSmax⁡Ce4pnDAHPS,e4pCpepnDAHPS,pepKDAHPS,e4p+Ce4pnDAHPS,e4pKDAHPS,pep+CpepnDAHPS,pep,

(A.33) $$\begin{array}{c} r_{\text{PGM}}=\frac{r_{\text{PGM}}^{\max}\left(C_{\text{g}6\text{p}}-\left(C_{\text{g}1\text{p}}/K_{\text{PGM},\text{eq}}\right)\right)}{K_{\text{PGM},\text{g}6\text{p}}\left(1+\left(C_{\text{g}1\text{p}}/K_{\text{PGM},\text{g}1\text{p}}\right)\right)+C_{\text{g}6\text{p}}}, \end{array}$$

(A.34) $$\begin{array}{c} r_{\text{G}1\text{PAT}}=\frac{r_{\text{G}1\text{PAT}}^{\max}C_{\text{g}1\text{p}}C_{\text{atp}}\left(1+{\left(C_{\text{fdp}}/K_{\text{G}1\text{PAT},\text{fdp}}\right)}^{n_{\text{G}1\text{PAT},\text{fdp}}}\right)}{\left(K_{\text{G}1\text{PAT},\text{g}1\text{p}}+C_{\text{g}1\text{p}}\right)\left(K_{\text{G}1\text{PAT},\text{atp}}+C_{\text{atp}}\right)}, \end{array}$$

(A.35) $$\begin{array}{c} r_{\text{MurSynth}}=r_{\text{MurSynth}}^{\max}, \end{array}$$

(A.36) $$\begin{array}{c} r_{\text{TrpSynth}}=r_{\text{TrpSynth}}^{\max}, \end{array}$$

(A.37) $$\begin{array}{c} r_{\text{MetSynth}}=r_{\text{MetSynth}}^{\max}, \end{array}$$

*Kinetics Parameters.* See Table 3.

## Nomenclature

*μ*: Specific growth rate or intracellular dilution rate
*D*: Dilution rate.

### Metabolite

Glc: Glucose
g6p: Glucose-6-phosphate
f6p: Fructose-6-phosphate
Fdp: Fructose-1,6-biphosphate
Gap: Glyceraldehyde-3-phosphate
Dhap: Dihydroxyacetonephosphate
Pgp: 1,3-Diphosphoglycerate
3pg: 3-Phosphoglicerate
2pg: 2-Phosphoglicerate
Pep: Phosphoenolpyruvate
pyr: Pyruvate
6pg: 6-Phosphogluconate
ribu5p: Ribulose-5-phosphate
xyl5p: Xylulose-5-phosphate
sed7p: Sedoheptulose-7-phosphate
xyl5p: Xylulose-5-phosphate
sed7p: Sedoheptulose-7-phosphate
rib5p: Ribose-5-phosphate
e4p: Erythrose-4-phosphate
Glp: Glucose-1-phosphate.

### Cometabolite

Atp: Adenosintriphosphate
Adp: Adenosindiphosphate
Nad: Diphosphopyridindinucleotide, oxized
Nadh: Diphosphopyridindinucleotide, reduced
Nadp: Diphosphopyridindinucleotide-phosphate, oxized
Nadph: Diphosphopyridindinucleotide-phosphate, reduced
Amp: Adenosinmonophosphate.

### Enzyme

ALDO: Aldolase
DAHPS: DAHPS synthase
ENO: Enolase
G1PAT: Glucose-1-phosphate adenyltransferase
G3PDH: Glycerol-3-phosphate dehydrogenase
G6PDH: Glucose-6-phosphate dehydrogenase
GAPDH: Glyceraldehyde 3-phosphate dehydrogenase
MetSynth: Methionine synthesis
MurSynth: Mureine synthesis
PDH: Pyruvate dehydrogenase
PEPCxylase: PEP carboxylase
PFK: Phosphofructokinase
PGDH: 6-Phosphogluconate dehydrogenase
PGI: Phosphoglucoisomerase
PGK: Phosphoglycerate kinase
PGM: Phosphoglucomutase
PGluMu: Phosphoglycerate mutase
PK: Pyruvate kinase
PTS: Phosphotransferase system
R5PI: Ribose phosphate isomerase
RPPK: Ribose phosphate pyrophosphokinase
RU5P: Ribulose phosphate epimerase
SerSynth: Serine synthesis
Synth1: Synthesis 1
Synth2: Synthesis 2
TA: Transaldolase
TIS: Triosephosphate isomerase
TKa: Transketolase a
TKb: Transketolase b
TrpSynth: Tryptophan synthesis.
